# Sustainable Elastomers for Actuators: “Green” Synthetic Approaches and Material Properties

**DOI:** 10.3390/polym15122755

**Published:** 2023-06-20

**Authors:** Olga V. Filippova, Aleksey V. Maksimkin, Tarek Dayyoub, Dmitry I. Larionov, Dmitry V. Telyshev

**Affiliations:** 1 Institute for Bionic Technologies and Engineering, I.M. Sechenov First Moscow State Medical University (Sechenov University), Bolshaya Pirogovskaya Street 2-4, 119991 Moscow, Russia; aleksey_maksimkin@mail.ru (A.V.M.); tarekzd@windowslive.com (T.D.); dmitry.larionov0625@gmail.com (D.I.L.); telyshev@bms.zone (D.V.T.); 2 Department of Physical Chemistry, National University of Science and Technology “MISIS”, 119049 Moscow, Russia; 3 Institute of Biomedical Systems, National Research University of Electronic Technology, Zelenograd, 124498 Moscow, Russia

**Keywords:** green chemistry, sustainable elastomers, bioresources, mechanical properties, soft actuators

## Abstract

Elastomeric materials have great application potential in actuator design and soft robot development. The most common elastomers used for these purposes are polyurethanes, silicones, and acrylic elastomers due to their outstanding physical, mechanical, and electrical properties. Currently, these types of polymers are produced by traditional synthetic methods, which may be harmful to the environment and hazardous to human health. The development of new synthetic routes using green chemistry principles is an important step to reduce the ecological footprint and create more sustainable biocompatible materials. Another promising trend is the synthesis of other types of elastomers from renewable bioresources, such as terpenes, lignin, chitin, various bio-oils, etc. The aim of this review is to address existing approaches to the synthesis of elastomers using “green” chemistry methods, compare the properties of sustainable elastomers with the properties of materials produced by traditional methods, and analyze the feasibility of said sustainable elastomers for the development of actuators. Finally, the advantages and challenges of existing “green” methods of elastomer synthesis will be summarized, along with an estimation of future development prospects.

## 1. Introduction

**Figure 1:**
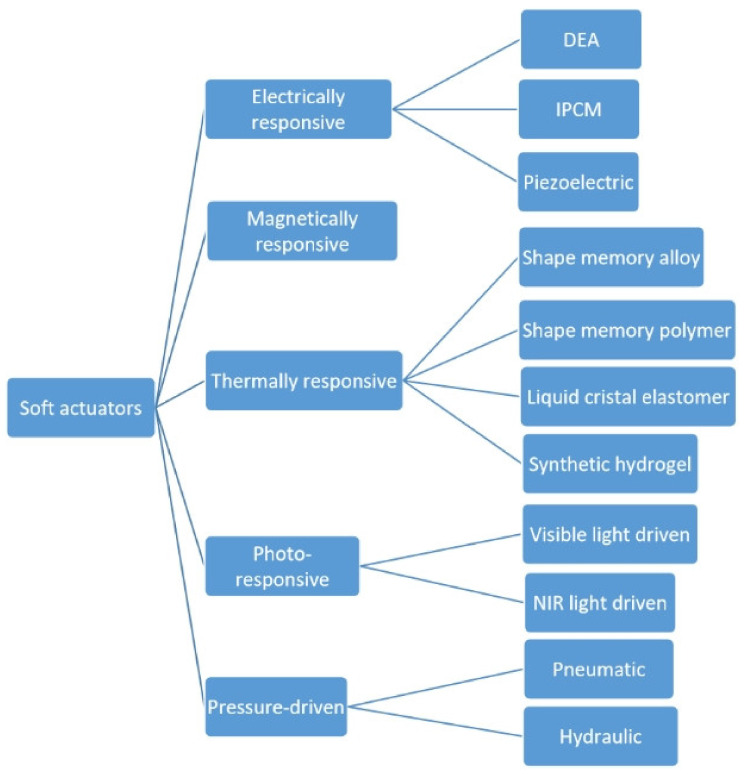
Different types of soft actuators (adapted from [32]).

The term “elastomers” covers a wide range of materials whose versatility provides almost unlimited potential for numerous applications. They are used in the manufacture of tires [1], coatings [2], membranes [3,4], and medical products [5,6,7,8]. Such popularity can be explained by their superior thermal, physical, mechanical, and electrical properties. It is also important that there are many tools to customize these properties, from synthesis to filler introduction, all of which make it easy to choose an elastomer with the properties needed for a particular application. The development of actuators is one of the fields in which elastomers play a crucial role. Polymer actuators are intended to replace traditional actuators, which are understood to be internal combustion engines and various types of electric motors. The idea is to create an efficient, noiseless, and compact device. Creating polymer actuators is a broad area of research that is not limited to elastomeric materials. There are many research trends in this field: pressurized fluid actuators [9], hydrogel-based actuators [10], shape memory alloy/polymers (SMAs/SMPs) [11,12,13], wearable actuators [14], electroactive polymer actuators [15,16], dielectric elastomer actuators (DEAs) [17,18,19], and actuators based on stimuli-responsive materials that could be triggered by temperature [20,21], light [22,23,24], pressure [25], pH [26], and magnetic [27] or electric fields [28,29,30,31]. Different types of actuators and materials used for their design are presented in Figure 1 [32].

Dielectric elastomer actuators (DEAs) have a special place in these studies: they have been intensively studied and developed since the 1990s, and considerable progress has been made in this area [33,34,35,36,37]. Of course, this has been accomplished owing to the specific properties of elastomeric materials, such as large deformation capability, low Young’s modulus, high dielectric strength, and hyperelasticity. It is very important that elastomers are able to alter their shape in response to electrical stimuli and store energy: this is the basis for the DEA development. DEAs have a number of advantages: large strain value (>300%), good power density and electromechanical conversion efficiency, and high response speed [38,39,40]. The main drawback of this actuator type is the high voltage needed for activation, which results in difficulties related to voltage breakdown possibility and miniaturization of the device. Two ways of decreasing activation voltage are enhancing dielectric permittivity and lowering the elastic modulus of the elastomer under study. These parameters should also be kept in mind during the development of new elastomeric materials.

The main elastomeric materials commonly used for actuator development are polyurethanes [41,42,43], silicones [44,45,46], and acrylic elastomers [46,47,48]. In some studies, tri-block copolymers were also analyzed: poly(styrene-b-ethylbutylene-b-styrene) (SEBS) [49,50], poly(styrene-b-butyl acrylate-b-styrene) (SBAS) [51], and poly(methyl methacrylate)(PMMA)-b-poly(n-butyl acrylate)(PnBA)-b-poly(methyl methacrylate)(PMMA) [52]. Each material type has its own advantages and disadvantages. At present, they are obtained by traditional synthesis methods, which often involve hazardous components. However, it is worth noting that these methods are exhaustively elaborated, and most of them have been long implemented in the industry.

Since the development of actuators is one of the steps towards the creation of artificial muscles as well as the production of rehabilitation medical devices, robotic surgical assistants, soft exoskeletons, i.e., many medical products that involve direct contact with humans, it is especially important to think about the safety of the materials from the very beginning of actuator development.

**Figure 2:**
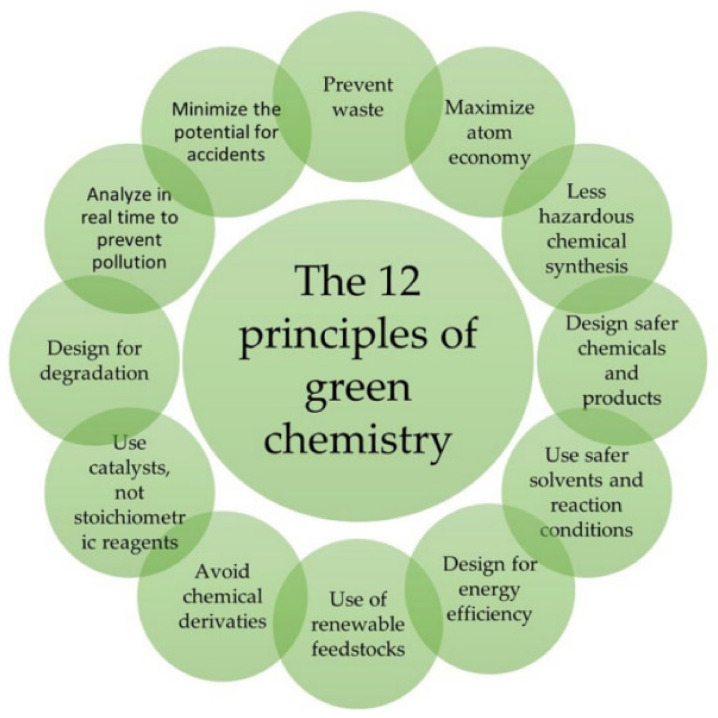
Scheme of the 12 principles of Green Chemistry [55].

For this purpose, one can consider green chemistry methods. Today many efforts are devoted to the creation, development, and improvement of the synthesis of sustainable polymeric materials. Twelve principles of green chemistry were formulated in 1998 [53]. Since then, they have undergone some modifications, but the basic ideas remain unchanged (Figure 2). Significant progress has been made regarding many items on the list over this period. M.A. Dubé and S. Salehpour, in their work [54], emphasized the most important theses for future development: “use less hazardous chemical synthesis”, “design safer chemicals and products”, “use safer solvents and reaction conditions”, and “use renewable feedstocks”.

In elastomer chemistry, there is also a trend toward switching to renewable resources, avoiding harmful organic solvents, and tending to perform synthesis under milder conditions.

In this review, current approaches to obtaining sustainable elastomers and minimizing environmental and human health hazards will be described. The properties and features of the resulting sustainable elastomers will be discussed. It will be evaluated if they can compete with materials produced by conventional methods. Additionally, when considering alternative green chemistry methods, we should always keep in mind the possibility of scaling them up. Based on this information, the feasibility of using sustainable elastomers in actuator design will be assessed.

We shall consider four groups of elastomeric materials: polyurethanes, silicones, acrylic polymers, and alternative materials. In each section, traditional methods of synthesis and their drawbacks with regard to their ecological impact will be discussed first, followed by a description of the existing green chemistry methods. Besides obvious achievements in this area, challenges and problems arising on this path as well as prospects for the development of sustainable elastomers synthesis will be considered.

## 2. Polyurethanes

**Figure 3:**
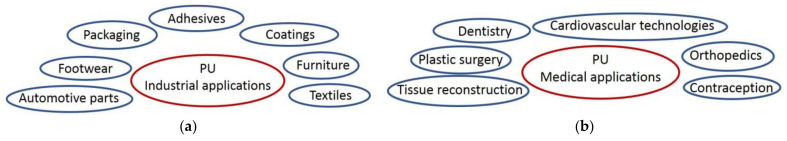
Main applications of PU in industrial (**a**) and medical (**b**) fields (adapted from [68]).

Polyurethanes (PUs) represent a large group of heterochain polymers with a wide range of physical, mechanical and chemical properties. Diverse composition and structure, different properties, and easy processing enable the production of these materials in a variety of forms and their application in different industrial [56,57,58] and medical fields [59,60,61,62,63] (see Figure 3).

Conventionally, polyurethanes are produced by a polycondensation process based on the reaction between isocyanate, polyol, and a chain extender at an elevated temperature [64]. The main reaction is the formation of the urethane bond, which occurs when an isocyanate reacts with an alcohol group of the polyol. The synthesis of polyurethanes is generally carried out using two methods [65]: either by mixing all the ingredients at the same time (“one-pot” technique) or by the preliminary reaction of polyol with isocyanate excess in order to form a “prepolymer”, followed by reaction with a chain extender to obtain a block copolymer with alternating hard segments (consisting of isocyanate and a chain extender) and soft segments (consisting of the polyol component) [66]. The hard segments impart mechanical strength, while the soft segments provide flexibility. The prepolymer method has been demonstrated to have certain benefits: higher structure organization and better control over resulting polymer properties. [67].

PUs can be classified into isocyanate (aliphatic, alicyclic or aromatic) or polyol (based on polyether, polyester or polycarbonate) types used for the synthesis. The nature of each component strongly influences the properties of the final product. For example, polyurethanes obtained using aliphatic diisocyanates have demonstrated greater resistance to UV exposure, whereas polyurethanes obtained using aromatic diisocyanates undergo photodegradation [69,70]. It has also been demonstrated that polyurethanes based on aromatic diisocyanates are less biocompatible than those based on aliphatic diisocyanates due to toxic degradation products [71]. The most commonly used isocyanates are 2,4-tolyenediisocyanate (TDI) and 4,4′-diphenylmethane diisocyanate (MDI) [72]. PUs produced on their basis differ significantly in structure and performance characteristics [73] as well as in ecological safety. It was shown that polyurethanes produced using toluene diisocyanate decompose under physiological conditions with the formation of 2,4-toluene diamine, which is known to be toxic [74]. Kääriä et al. [75] carried out an in vivo study using polyurethane prepared from aromatic 4,4′-methylenediphenyl diisocyanate and observed cytotoxicity due to the aromatic amine 4,4′-methylenedianiline formed in polymer degradation.

Polyols are usually dihydroxyl-terminated polyethers, polyesters, and polycarbonates with a molecular weight varying in the range from 1000 Da to 5000 Da. Molecular weight and polyol type play a significant role in the physical, chemical, and mechanical properties of resulting polyurethane. For example, polyester-based polyurethanes have good mechanical strength and thermal stability, but they are susceptible to hydrolysis [76].

Chain extenders are low molecular weight diols or diamines, and their role is to increase the block length of the hard segment. The structure of the chain extenders determines the rigidity of the hard blocks and density of hydrogen bonds [77].

The main types of components used in PU synthesis are presented in Table 1.

It has been reported [79] that many materials, including PUs, exhibit electrostrictive properties, or, in other words, the ability to deform under the application of an electric field. This observation has drawn significant attention to these materials as a new class of electromechanical materials. From this point of view, it is also important that PUs provide benefits such as flexibility, softness, high dielectric permittivity, and the ability to be cast into various shapes. Superior dielectric and mechanical properties as well as outstanding actuating behavior make polyurethanes suitable materials for actuators and sensors [80]. The important advantage of PUs over conventional elastomers is the simplicity of their polymeric chains design by changing the proportion of hard and soft segments. To optimize the properties of PU materials essential for application as soft actuators and even give some additional features, such as biocompatibility, self-repair properties, and shape memory, one could vary the composition of these blocks or add other components. Electroactive polyurethane-based actuators have shown their potential in soft robotics development due to their large flexural strain, fast response, and good durability [81].

However, the isocyanates-associated toxicity is still a serious problem on the way of expanding the use of PUs as biomaterials or as hybrids with biopolymers. Not only are isocyanate reagents themselves and their degradation products toxic, but they are also derived from the extremely poisonous and reactive phosgene gas; in the industry, isocyanates are produced by the reaction of amines with phosgene [82], and large amounts of corrosive hydrogen chloride are produced as a side product in this process. Numerous studies have been carried out focusing on the elimination of phosgene from the synthesis of isocyanates [83,84,85,86]. Continued use of highly hazardous isocyanates in PU synthesis can adversely affect the health of personnel involved in handling and production. Any unreacted residue remaining in PU may also have a negative impact on the health of end users [87]. For example, tin-based catalysts, such as dibutyltindilaurate, which remain after polymerization, increase the toxicity of PUs. This also restricts their use in bio-material applications. From the perspective of green chemistry, the most important direction in polyurethane synthesis is the reduction/avoidance of isocyanates as the most hazardous ingredients. Thus, there is a critical need to develop new processes and new sustainable PU materials that either do not involve isocyanates or require less of it.

There are two main approaches to obtaining sustainable polyurethanes:

(1) The reduction of the amount of toxic isocyanates by using mixtures with less hazardous isocyanate types and using polyols and diisocyanates derived from renewable resources;
(2) The complete elimination of isocyanates—the development of non-isocyanate polyurethanes (NIPU).

### 2.1. Reduction of Amount of Toxic Isocyanates

The authors in [88] exploit the idea that the use of biobased diisocyanates with lower volatility instead of petrochemical ones may have a positive effect on the environment and will lead to biobased PUs. So, in this work partial replacement of petrochemical diisocyanates with biobased diisocyanates is represented by using two types of diisocyanate mixtures: aliphatic-aliphatic, based on hexamethylene diisocyanate with partially biobased aliphatic diisocyanate (Tolonate™ X FLO 100 (HDI-FLO), and aromatic–aliphatic, based on diphenylmethane diisocyanate with partially biobased diisocyanate (MDI-FLO). This results in a reduction of 25 wt. % of petrochemical diisocyanate. Partially biobased PUs were prepared using the prepolymer method. In the second stage, biobased 1,3-propanediol was used as the chain extender and dibutyltin dilaurate as the catalyst. As we discussed earlier, the use of tin-based catalysts is one of the drawbacks of the polyurethane synthesis process and does not comply with the principles of green chemistry. The thermal, thermomechanical, mechanical, and physico-chemical properties of PUs based on two types of diisocyanate mixtures were investigated and compared. The mechanical properties of the obtained PU elastomers are presented in Table 2.

The authors concluded that HDI-FLO-based PU demonstrates better thermal and mechanical behavior. However, from the perspective of using these materials as soft actuators, the results are not so obvious, because MDI-based PU has a lower Young’s modulus, while HDI-based PU has higher elongation at break.

The same scientific group continued this research and chose HDI-FLO-based PU as a better sample and replaced petroleum-based polyol with biobased polyester [89]. They also used the solvent-free prepolymer method with the addition of a tin-based catalyst in the second stage, but changed the chain extender—in this work, they used biobased 1,4-butanediol. The properties of PUs obtained were compared to MDI-based PU. Two parameters: tensile strength and elongation at break, were measured as the representative values of the mechanical properties. Biobased diisocyanate strongly influences the thermal and thermomechanical properties of PU. For reference material, tensile strength was in the range of 9.2–25.8 MPa, and elongation at break was in the range of 218–803%, depending on the hard segment content in the polymer structure. The ranges of these parameters for biobased samples were 6.5–33.3 MPa and 421–904%, respectively. A total of 50% of biobased diisocyanate was considered as the optimal amount in the diisocyanate mixture that results in material with good mechanical properties.

The influence of polyol characteristics and chain extender type on PU properties was also studied [90]. Biobased poly(propylene succinate)s were synthesized at different temperatures and used as polyols in PU synthesis. This process was carried out with MDI as diisocyanate and 1,4-butanediol and 1,3-propanediol as the chain extenders of natural origin. It should be noted that the synthesis of PU by this process can be carried out without a tin-based catalyst. The chemical structure, thermal, thermomechanical, mechanical, and physical properties of synthesized biobased PU were analyzed. It was found that the type of chain extender influences the properties of PU more compared to the conditions of polyol synthesis. The best samples under investigation had a low glass transition temperature in the range of 0–5 °C and good mechanical properties: tensile strength in a range of 17–30 MPa, elongation at break in a range of 438–591%. Moreover, it was proven that the measured samples are characterized by low stiffness and good ability regarding damping capacity.

Miao, S. et al. [91] studied soybean oil-based PUs as possible biomaterials. They synthesized polyols from soybean oil by ring-opening reaction with lactic acid. HDI was used as diisocyanate. The final reaction of PU synthesis was carried out without a tin-based catalyst. Biobased PUs with a tensile strength of 9.30–27.1 MPa and elongation at break of 74.1–110.7% were prepared. The latter values are rather low, which means that the material is rigid and not able to deform to a great extent. The biocompatibility test with mouse fibroblast cells showed good adhesion and growth behavior, indicating its potential application as a biomedical material. Various diols derived from vegetable oils, HDI, and a corn sugar-based chain extender were used for two-step polymerization without a catalyst [92]. Different compositions of hard and soft segments were synthesized, resulting in a wide range of material properties. Elongation at break varied from 18% to 900%, and Young’s modulus from 14 MPa to 319 MPa. This variety enables one to choose desirable properties for different practical applications. In a review published in 2014, different approaches to biobased diisocyanates from vegetable oils are discussed [93].

Another source of biobased polyols was suggested by Jung, Y.S. et al. [94,95]. They used fermented corn for the synthesis of polyol, two types of diisocyanates: aromatic MDI and aliphatic (bis(4-isocyanatocyclohexyl) methane, H_12_MDI, and biobased chain extenders PDO [94] or BDO [95] for a solvent-free, one-shot procedure of PU synthesis. A tin-based catalyst was needed in this process. The properties of PUs obtained are presented in Table 3 and Table 4. In both cases, the use of aromatic MDI resulted in better thermal and mechanical properties for biobased PU. This means that the isocyanate type influences the composition of PUs in terms of hard/soft segments ratio and distribution, which in turn affects the microphase separation that defines many properties of material. Future investigations into these biobased PUs could be devoted to their possible application as shape memory polymers in 3D/4D printing for soft actuators development. A similar process for the synthesis of PUs based on aliphatic diisocyanate and biobased polyol was studied in [96], and the obtained samples were analyzed from the perspective of their potential application as filaments for 3D printing to make a soft actuator. Very promising mechanical properties were estimated: low Young’s modulus in the range of 2.4–4.4 MPa and large elongation at break in the range of 900–1800% were obtained. Hardness Shore A was between 74 and 85.

A recent article [97] is devoted to the development of biobased shape memory PUs for application in 3D/4D Printing Filaments. Polyols derived from corn oil were used as building blocks for soft segments and the MDI and chain extender BDO for hard segments. The mechanical properties obtained for the samples are promising for the future employment of these partially biobased PUs as shape memory materials in 3D printing.

A comprehensive study was performed by another group [98]. They used vegetable oil-based aliphatic diisocyanate: 1,7-heptamethylene diisocyanate (HPMDI), petroleum-based polyester macrodiol, and four different chain extenders. Three types of polycondensation techniques: one-shot method, prepolymer method, and multistage polyadditional method, were studied. Dimethylformamide as solvent and tin octanoate as catalyst were used in each type of process. Effect of hard segments content on properties of resulted polymers were investigated and compared to known data for PU based on petroleum-derived diisocyanates. A wide range of properties were estimated: elongation at break varied from 6.8% to 758%, Young’s modulus varied from 62 MPa to 420 MPa, and tensile strength from 8.9 MPa to 31.4 MPa. The main idea of this work is that it is possible to tune mechanical properties by controlling the behavior of hard segments crystallization, which strongly depends on a synthetic approach.

An interesting attempt to improve the mechanical properties of PU elastomers based on aliphatic diisocyanate HDI was made in [99]. Due to the poor mechanical properties, the application of PU elastomers based on biodegradable poly(Ɛ-caprolactone) (PCL) and low-toxicity aliphatic diisocyanates in biomedical field is limited. The idea was to use a stereocomplex formation between the PU elastomer and polylactide. Macrodiols based on PCL and PLA served as building blocks for soft segments, and HDI and chain extender BDO for hard segments. Samples characterized in high values of elongation at break (760–1020%) and rather low values of Young’s modulus (12–23 MPa) were obtained.

The regulation of thermal and mechanical properties was performed by using isosorbide and 1,4-butanediol [100]. Biobased isocyanate derived from fatty acids was used for the synthesis of hard segment-containing blocks. It was found that increasing isosorbide content resulted in poorer mechanical properties: elongation at break decreased ten-fold from 350% to 30% for the samples with 5 wt. % and 20 wt. % of isosorbides, respectively.

### 2.2. Non-Isocyanate Polyurethanes

**Figure 4:**
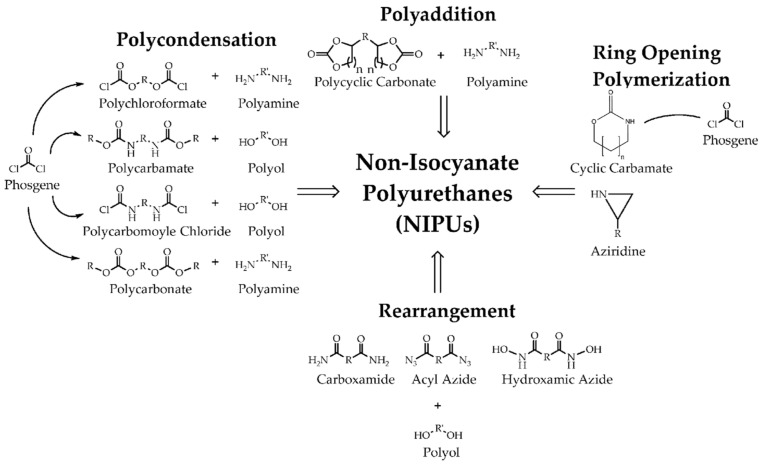
Main routes for NIPUs synthesis [106].

Different approaches to the synthesis of diisocynate-based PUs from renewable resources were considered in the previous section. However, the presence of residual amounts of isocyanate in PU still poses a hazard to human health and risk to environment. As an alternative, non-isocyanate PUs (NIPUs) were suggested. The first successful attempt was published in 1957 by Dyer and Scott [101]. Wilkes [102] developed this method and applied it to renewable resources. Since then, numerous research has emerged in this field. The first industrial application of NIPUs was realized by Nanotech Inc. under the trade name Green Polyurethane TM. Many reviews are devoted to the different methods of NIPU synthesis. The four main routes of NIPUs synthesis are formulated (see Figure 4): ring-opening polymerization, rearrangement, polycondensation, and polyaddition. They are extensively described in the literature [103,104,105]. As one can see from the scheme presented in Figure 4, three of these routes involve highly toxic phosgene, aziridine, or acylazides, or result in the generation of corrosive side-products such as HCl. Therefore, their utilization has no advantages from an environmental point of view. Thus, a more promising way is the polyaddition process using cyclic carbonates and diamines or polyamines, which allows for the elimination of isocyanate from the synthesis route and broadens the use of biobased renewable resources in products such as vegetable oils, fatty acids, starch, sugar, etc.

Unfortunately, almost all the research is devoted to the development of different green synthetic methods and only a few of them describe the mechanical or electrical properties of final NIPUs.

A recent study [107] describes the synthesis of tailor-made biobased NIPUs from triglycerides previously carbonated in scCO2 and three different diamines. The main goal was to control the cross-linking density of NIPUs using water or bio-alcohols as capping compounds. This would make it possible to precisely adjust the physical properties of a material to meet the requirements of its desired application. The results of the mechanical property measurements are presented only in a graphical way, so only approximate evaluation is possible. The obtained values of tensile strength, elongation at break, and Young’s modulus are plotted against the functionality of cyclic carbonated or carboxilated soybean oil used in polymerization. All parameters are characterized by low values: tensile strength is up to 5 MPa, elongation at break is less than 140%, and Young’s modulus for three of four samples is low in PUs context—it does not exceed 5 MPa, and only for one sample the value reaches 25 MPa.

The synthesis of polyester-based NIPUs and an evaluation of the possible biomedical applications are presented in [108]. A non-isocyanate high molecular weight polyurethane was obtained from 1,6-hexanedicarbamate and polycarbonate diols using a transurethanization reaction. The highest molecular mass achieved was 58,600 Da. Polymers were characterized by NMR, FTIR, HPLC, and DSC. The molecular mass of polycarbonate-based diols was varied from 500 Da to 2000 Da in order to obtain a different length of soft segments. So, the lowest glass transition temperature (−41 °C) was obtained with the long-chain diol (2000 g/mol). Fibrous scaffolds made of obtained NIPUs using the electrospinning technique were investigated. They can be used for cardiac tissue development as a biomimetic material, replacing load-bearing pericardial tissue. For these purposes, the evaluation of cytotoxicity is obligatory. Bare mats and biofunctionalized collagen mats were studied. It was found that the properties of the obtained samples were comparable with those of collagen mats. Bare mats had no negative effect on cell proliferation, which makes these NIPUs promising candidates for tissue bioengineering.

The composition of NIPUs strongly influences thermal and mechanical properties. To investigate this relationship, NIPUs were synthesized from carbonated soybean oil and either short diamines or oligomeric amides derived from fatty acids [109]. It was found that the materials exhibited low mechanical properties, which was attributed to cross-linking when short diamine is used. NIPUs with improved elongation break were synthesized with the use of oligomeric amides. The dependence of tensile strength on the cyclic carbonate content of soybean oil is also discussed. However, it should be noted that a good combination of mechanical properties—low Young’s modulus and large elongation at break—needed for soft actuators was not achieved for any sample under study.

It is a promising approach to develop hybrid NIPUs crosslinked with other functional systems. One example of such a system is the hybrid of polyhydroxyurethane and starch with improved mechanical and structural characteristics [110]. The first step involved the synthesis of crystallizable polyhydroxyurethanes (PHUs) through the non-isocyanate and catalyst-free route with step growth polymerization of ethylene carbonate and three different diamines (1,2-ethanediamine, 1,4-butanediamine and 1,6-hexanediamine). Then, PHUs were combined with gelatinized starch (HAGS) to synthesize HAGS/PHU hybrid materials. Hydroxyl groups of HAGS and PHUs were found to predominantly contribute to intermolecular hydrogen bonding. The films produced using the HAGS/PHU hybrid materials exhibited tunable mechanical properties, with a tensile strength ranging between 1.7 MPa and 3.2 MPa and an elongation at break varying between 45% and 121%. These routes of synthesizing non-isocyanate polyurethanes and their hybridization will open a pathway to develop novel hybrid materials with many more natural biopolymers.

Fully biobased non-isocyanate polyurethane-based materials could become a sustainable platform to produce piezoelectric materials [111]. NIPUs with ferroelectric properties could be successfully synthesized using a solvent-free reactive extrusion process based on an aminolysis reaction between resorcinol bis-carbonate and different diamine extension agents. Structure−property relationships were established, indicating that the ferroelectric behavior of these NIPUs depends on the nanophase separation inside these materials. The molecular architecture of the obtained NIPUs has been designed to show a final polar structure and, consequently, obtain the ferroelectric-like behavior. Using resorcinol bis-carbonate and cadaverine as monomers, a NIPU characterized by high Tg and a permanent dipolar moment has been obtained. Macroscopic ferroelectric switching was achieved, and it was found that piezoelectric activity can be tuned by changing the NIPU molecular architectures. The dielectric properties of the NIPUs were characterized by measuring their complex dielectric permittivity as a function of temperature.

New approaches to the synthesis of sustainable NIPUs are constantly emerging. Many articles and reviews deal with the synthesis and characterization of the structure of such polyurethanes. This field is being actively developed due to the increasing attention to environmental issues and the demand for a transition to green synthesis methods. It was shown that NIPUs exhibit improved thermal stability, higher resistance to nonpolar chemical solvents, increased adhesion, and wear resistance compared with traditional PUs [112,113]. However, the mechanical and electrical properties of NIPUs have only been analyzed in a few articles; therefore, it is difficult to conclude to what extent the properties of the obtained polyurethanes are close to those of conventional ones. This is currently a gap in the field. In terms of green chemistry, phosgene-free and non-isocyanate polyurethanes are the most promising group for which the most hazardous precursors have been eliminated. Those synthetic methods that do not require the use of a tin-based catalyst or elevated temperature have an additional advantage. Further studies on the mechanical and electrical properties of polyurethanes would be highly useful in the development of soft actuators.

The typical properties of traditional polyurethane elastomers suitable for use in soft actuators are summarized in the Table 5. For the moment, only these parameters can be used to compare sustainable and traditional polyurethanes. While the mechanical properties (mainly three parameters: tensile strength, elongation at break, and Young’s modulus) have been investigated in several articles, electrical properties have hardly been studied. The analysis of the literature data enabled us to conclude that using the first approach: the reduction of use of the most toxic diisocyanates in synthesis and the increase in biobased content in the final polymer using biobased polyols and chain extenders, one can obtain polyurethanes with properties close to those of conventional ones. In fact, they could already be used as a basis for the development of actuators. However, this compromise is not so attractive in terms of environmental considerations. The main perspective in this field would be the combination of green chemistry approaches, characterization and optimization of the electrical and mechanical properties of the resulting sustainable polyurethanes, and the development of actuators based on such sustainable polyurethanes.

## 3. Silicone Elastomers

**Figure 5:**
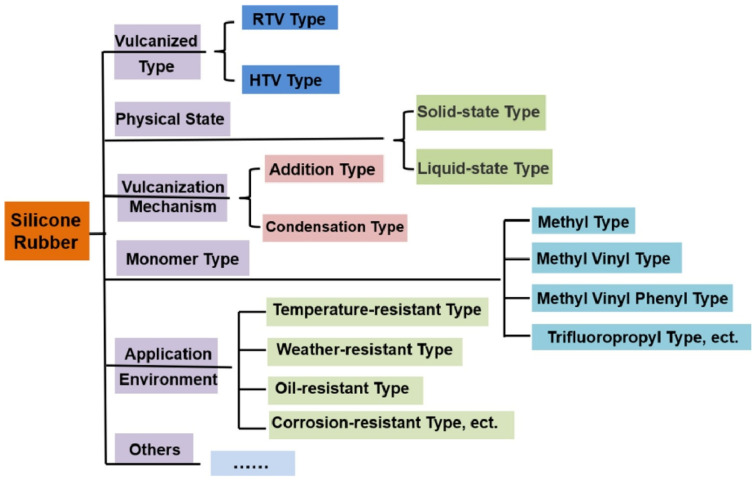
Classification of silicone rubbers (adapted from [127]).

Silicone rubbers are elastomeric materials that have become practically indispensable in many application fields, such as the automotive industry, construction, electronics, food processing, and medicine [116,117,118,119]. Silicone elastomers have been used in numerous scientific and practical studies related to the development of artificial muscles, soft robotics, and actuators [120,121,122,123]. Their superior chemical, thermal, and mechanical stability as well as their good biocompatibility have made silicone elastomers very popular in biomedical engineering [124,125,126]. Silicone rubbers can be divided into many groups according to different parameters (see Figure 5).

Industrial synthesis of silicones is based on the reaction of chlorosilanes with water. In this process, a great amount of corrosive and harmful hydrogen chloride is formed. The use of chlorosilanes does not meet the requirements of green chemistry principles, so many scientific studies have specifically focused on the search for alternative monomers [128,129]. Alkoxysilanes were found to be the main substitute. In this case the acetic acid is formed, which is less aggressive. The drawback of this process is the slower curing rate.

The final stage of the synthesis of silicone rubber with a required set of physical properties is the curing process. There are three main types of curing agents used for silicones:

(1) Platinum-cured systems (addition-cured silicones)

**Figure 6:**
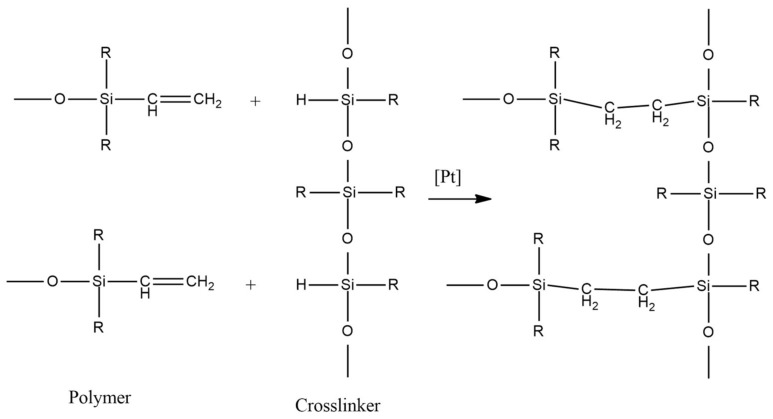
Reaction scheme for platinum-cured silicones.

This type of curing is based on hydrosilylation reaction catalyzed by Pt-complex (see Figure 6). One important advantage of this process is the absence of hazardous volatile side products. The possibility of controlling the curing speed by temperature is also valuable. The main drawback is the high probability of catalyst inhibition even by small amounts of catalyst poisons in the ambient air, especially amine- and sulfur-containing compounds.

(2) Tin-cured systems (condensation-cured silicones)

**Figure 7:**
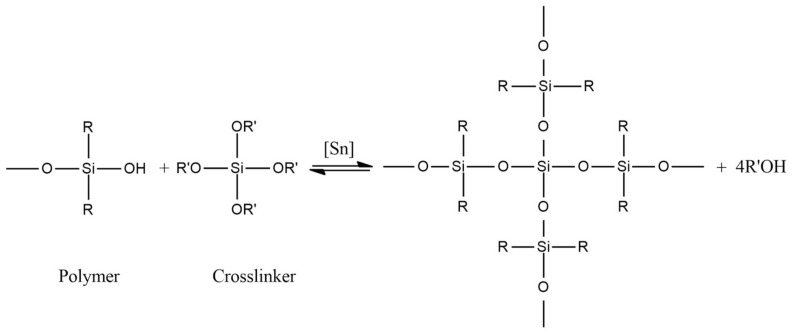
Reaction scheme for tin-cured silicones.

In this case, the reaction occurs between α, ω-dihydroxypolydimethylsiloxanes and silicic acid esters (see Figure 7). Typical catalysts for condensation curing are dibutyltin dilaurate and dibutyltin dioctoate. Water has a strong accelerating effect on the reaction rate, whereas temperature change has almost no influence. Shrinkage of the final elastomer caused by alcohol release and evaporation during the curing process can be mentioned as a drawback of this technique.

(3) Peroxide-cured systems (high temperature vulcanized silicones)

**Figure 8:**
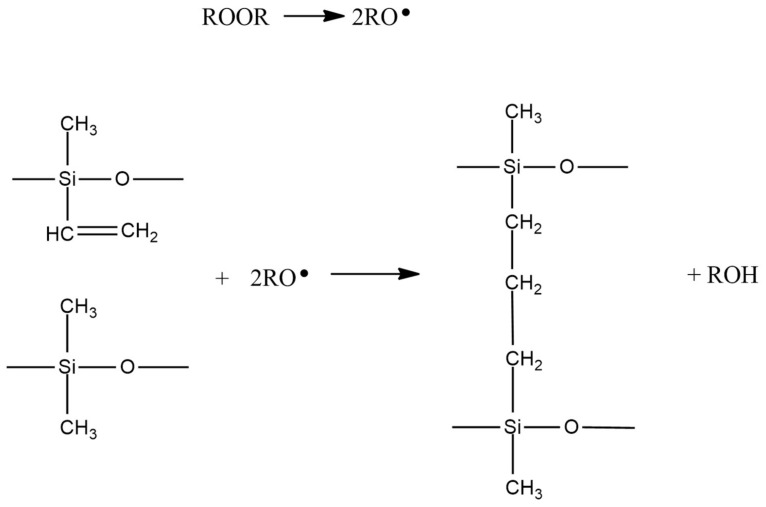
Reaction scheme for peroxide-cured silicones.

This is a free-radical process, initiated by the thermal decomposition of specific compounds (see Figure 8). Various organic peroxides may serve as free-radical generators for initiating this reaction. The advantage of this curing type is low sensitivity to catalyst poisons. Two main drawbacks are the elevated temperatures needed for the process and the formation of odorous volatile by-products.

An eco-friendly UV-curing process is now under study [130,131]. UV-curing is a fast process, which can be performed at a low temperature. It is especially important when temperature-sensitive substrates or components are used.

Pt-cured silicone elastomers are often used in the production of pharmaceutical, biotechnology, and food and beverage products. Tin-cured silicones are most commonly used to make flexible molds that can be used for casting polyurethane, epoxy or polyester resins, waxes, and low-temperature metals. They are not FDA compliant for food or skin applications because of residual amounts of tin. Peroxide-cured silicone sheets and tubing are more cost effective than platinum-cured ones because raw materials are cheaper.

Silicone elastomers are ideal candidate materials for dielectric actuators (DEA) development due to their high efficiency, reliability, and excellent mechanical properties, especially very low Young’s modulus and high flexibility. They are commercially available with a wide range of properties. Their main disadvantage is low values of dielectric permittivity, resulting in high operating voltages.

What are the main challenges of silicone elastomer production from an ecological point of view? The following issues do not comply with green chemistry principles: high energy consumption, lack of renewable bioresources usage, the high costs of Pt-based catalysts, the environmental hazard of tin-based catalysts, and high temperatures needed for the peroxide curing process.

**Figure 9:**
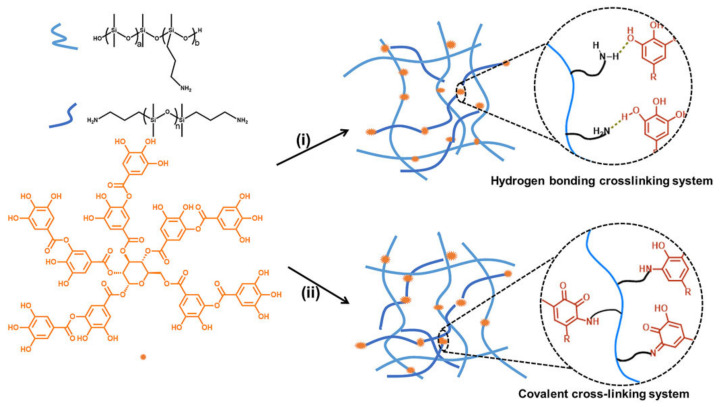
Preparation of silicone elastomers crosslinked by hydrogen bonding or covalent bonding. (i) H_2_O/solvent, room temperature; (ii) H_2_O/solvent, room temperature; 150 °C, 1 h [132].

The search for biobased or natural cross-linkers is a path to more sustainable silicone elastomers. S. K. Sen et al. [132] suggested the use of tannic acid as a green and natural crosslinker under a catalyst-free method for poly(aminopropylmethylsiloxane-co-dimethylsiloxane) as the base polymer. Crosslinker behavior can be switched from hydrogen bonding to covalent bonding by changing the temperature during curing reaction (see Figure 9). It was found that the mechanical properties of elastomers can be tuned by:

- The processing technique;
- The amount of tannic acid and aminopropyl-terminated polydimethylsiloxane;
- The molecular weight of the base polymer and content of -NH_2_-groups in it.

Materials obtained were characterized in low Young’s modulus: from 0.6 MPa to 5.1 MPa, with the highest value of elongation at break being 203%. These elastomers were used as adhesives in order to investigate their potential application.

Another type of natural cross-linker was studied by R. Bui and M. A. Brook [133]. They used the dimer of vanillin, which acted as a chain extender to form copolymers and/or a cross-linker to obtain elastomers from linear polymers. As the starting prepolymers, α,ω-(3-aminopropyl)polydimethylsiloxanes were used. Due to the low solubility of divanillin and slow reaction rate, the process was carried out at reflux in toluene, so it is difficult to consider this approach as a green method. The attempt to perform this synthesis without solvent resulted in very long reaction times (6 days) even at elevated temperature. The authors noted that one of the future goals should be to search for green catalysts for this process. The mechanical properties of the final elastomers can be controlled by the number of NH_2_-groups in the starting polymer. The range of elongation at break was 103–255% and the range of Young’s modulus was 0.1–3 MPa. It is interesting that for some samples, the hardness values of 31 and 54 on a 00-scale were obtained. Only a few types of commercially available silicone elastomers have such low values of hardness. One more feature of the obtained elastomers is the possibility to reprocess and reuse them easily due to their dynamic bond exchange mechanisms.

High energy input needed for silicone elastomers production can be reduced by obtaining the composite materials with acrylated soybean oil [134]. Very effective catalyst-free aza-Michael reaction in the absence of solvents allows for linking between aminoalkylsilicones and acrylated soybean oil. The main idea was to “dilute” the silicone elastomer without deteriorating its properties. The resulting silicone composites were described by hardness, surface energy, and thermal stability. The obtained materials are more sustainable than pure silicone elastomers because less silicone is required for a given application, and the products readily undergo degradation to silicone oils by base hydrolysis of the esters, which should facilitate recovery and reuse.

A similar approach was used in [135]. The authors also noted that silicones are expensive due to the energy-intensive process required to produce them. They proposed that the environmental impact of silicones could be reduced by diluting them with renewable materials such as triglycerides. It was established that elastomers with tunable Young’s modulus values could be formed with a soybean oil content of up to 76%. The incorporation of significant quantities of the soybean oil products in a silicone improves the sustainability of the synthetic polymer.

Research combining the synthesis and evaluation of the mechanical, electrical, and actuation properties of sustainable silicone elastomers was performed by A. Bele et al. [136]. Environmentally friendly methods were used to synthesize siloxane copolymers and, subsequently, to cross-link them into elastomers. Soft polar silicone-based elastomers were obtained using a three-step procedure. In the first stage, a series of copolymers with vinyl groups attached to the main backbone were synthesized by heterogeneous cationic ring opening polymerization. In the second stage, chloropropyl groups were grafted to the main backbone of the siloxane copolymer by UV-induced thiol-ene addition. Chloropropyl groups were chosen due to their ability to provide better thermal stability to the final polymers and a high dipole moment. In the third stage, copolymers with chloropropyl groups were cross-linked with an excess of tetraethyl orthosilicate and DBTDL as the catalyst. This condensation reaction occurs at room temperature in mild conditions with ethanol as the main by-product. The properties of the final elastomers in comparison with two commercial silicone elastomers are presented in Table 6.

As one can see from the table, dielectric elastomers with higher values of dielectric permittivity were obtained. There was also a significant decrease in Young’s modulus, which the authors attributed to the plasticizing effect of the alkyl substituents in the side chloropropyl groups. They concluded that these changes were induced by the chloropropyl groups, resulting in large out-of-plane actuation strains of 53% and 61%. It should be noted that values of elongation at break are quite low in comparison with reference materials.

In all the studies discussed, elastomers with a low elastic modulus were obtained, which is an advantage for actuators development. It was shown that sustainable copolymers with higher values of dielectric permittivity could be obtained by green chemistry methods. This is another parameter that allows one to lower the voltage needed for actuation. Therefore, these are very promising approaches and could result in more sustainable silicones with desirable mechanical and electrical properties. High values of elongation at break have not yet been reached in the studies discussed. Nevertheless, the values obtained for the sustainable silicone samples are comparable with some commercially available types, for example, PDMS Sylgard 184.

The mechanical parameters for typical silicone elastomers, that are widely used for soft actuators design are presented in Table 7 as an example.

## 4. Acrylic Elastomers

The third class of elastomers, which will be considered in this review, is acrylic elastomers. Due to their wide property range, they have found commercial applications in a large variety of fields, such as automotive non-tire applications, paints, coatings, textiles, and adhesives [139,140]. Other industries include the manufacturing of cement, caulk, sealants, ceramic, and paper [141,142]. The agricultural field also uses some types of acrylic elastomers [143].

**Figure 10:**
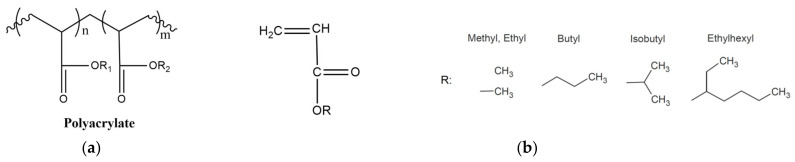
(**a**) Polyacrylate structure (**b**) common acrylic monomers.

The typical structure of a polyacrylate copolymer and the most common monomers are presented in Figure 10.

**Figure 11:**
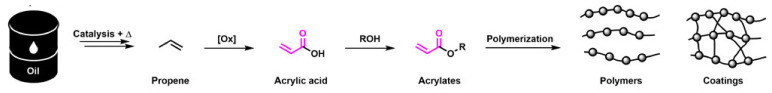
Traditional petrochemical-based synthetic approach to polyacrylates.

In the multistep processes typically used today, middle- and long-chain hydrocarbons are converted into olefins by cracking, then oxidized and derivatized into acrylates, which are subsequently polymerized to produce a wide range of plastics, resins, and coatings [144,145]. This scheme (see Figure 11) reflects the fossil origin of polyacrylates. Most research in the area of green chemistry of acrylates is focused on the replacement of fossil-based monomers with biobased ones. Numerous reviews and research articles [146,147,148,149,150,151,152,153,154] are devoted to the different bio-alternatives of acrylic monomers. Two large groups mentioned in a number of studies are cellulose-derivative acrylates obtained by esterification and vegetable oil-derived acrylates (including palm, olive, peanut, rapeseed, corn, and grapeseed oils) [147]. Other suggested sources are terpenes [148], lactic acid [149], vanillin [150], isosorbide [151], glycerol [152], fatty acids [153], and lactones [154]. These approaches described in the literature are an effective way to substitute petroleum-based monomers, but they have serious drawbacks in terms of green chemistry. Although the monomers are actually biobased, the synthetic routs require using fossil-based chemicals and solvents, which may pose serious health hazards. Among the methods proposed, enzymatic techniques and the selective reduction of itaconic acid can be considered as true green processes.

The main polymerization techniques currently used for polyacrylate synthesis are living anionic polymerization [155,156], controlled radical polymerization (mostly atom transfer radical polymerization (ATRP) [157], single-electron transfer-living radical polymerization (SET-LRP) [158,159], and reversible addition−fragmentation chain transfer polymerization (RAFT) [160] methods), emulsion radical polymerization [161], and photopolymerization [162]. The first two methods allow one to produce well-defined polymers with different architectures and are usually used for scientific purposes in order to create materials with specific structures and properties. The main ecological drawbacks of living anionic polymerization are the use of metalorganic catalysts, such as butyllithium, or hazardous solvents. Therefore, the main focus here is the replacement of toxic initiators and catalysts and the switch to green solvents or bulk polymerization. The great advantage of controlled radical polymerization methods is the possibility of polymerizing the different types of biobased monomers mentioned earlier as substitutes for fossil-based acrylic monomers, such as acrylic elastomers from lignin, cellulose, soybean oil, lysine, itaconate, furfurol, terpene, which may be produced by a combination of these methods, and click-chemistry or ring-opening polymerization. Various green solvents were also studied in order to replace traditionally used ones [163]; for example, water, ionic liquids, supercritical carbon dioxide (scCO2). Therefore, controlled radical polymerization represents a very important technique, which enables the sustainable production of polyacrylates from non-fossil feedstock. It should be noted that among the three mentioned techniques, ATRP implies the utilization of metal-based catalysts, which in residual amounts may be hazardous; the other two methods do not have this disadvantage. Emulsion and photo-polymerization are widely applied in the industry to obtain polyacrylates. These two techniques are inherently regarded as “green” methods. Photopolymerization can be carried out without solvent at room temperature. It is a very fast and efficient process. Emulsion polymerization is performed in water, which eliminates the use of organic solvents. In order to improve the sustainability of these processes, the main focus should be the use of renewable raw materials.

Acrylic elastomers are widely used in the development of soft actuators because of their large strain values. The most popular type used in this field is VHB™ 4910 manufactured by the 3M company [164]. As a polar elastomer, it has a high dielectric permittivity of about 4.7. In addition, it shows high elongation at break (more than 600%) and elastic modulus in the range of 0.4 to 2.3 MPa, according to reported data. It is produced in the form of a film, with an adhesive layer on both sides that facilitates the design of actuators. However, long response times after stress application and hysteresis during cycling can be noted as drawbacks.

**Figure 12:**
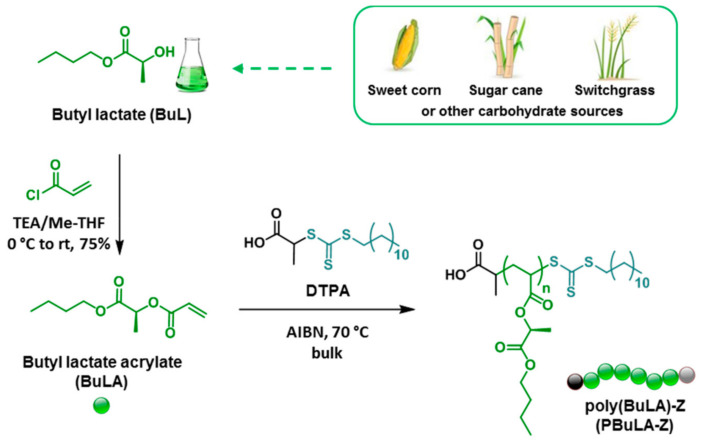
Synthesis of BuLA from biorenewable BuL solvent and their bulk reversible addition–fragmentation chain transfer (RAFT) polymerization [165].

Different groups of renewable acrylate raw materials used for elastomer synthesis will be discussed below. Solvent butyl-L-lactate was used to synthesized butyl-L-lactate acrylate, which was further polymerized into poly-butyl-L-lactate acrylate (see Figure 12) and copolymerized with isosorbide- and vanillin-derived acrylates using the RAFT method [165]. The middle block of triblock copolymers consisted of poly-butyl-L-lactate acrylate, which served as a soft segment, and end blocks that consisted of either isosorbide- or vanillin-derived polyacrylates, which served as glassy segments. Green solvent PolarClean was used to perform polymerization processes. Elongation at break and tensile strength measured for triblock copolymers was 250% and 0.83 MPa, correspondingly. The adhesive properties of copolymers were competitive with those of commercial pressure-sensitive adhesives. A promising approach to obtaining sustainable polyacrylates from alkyl-lactates is described in [166]. Cu(0) wire catalyzed SET-LRP in alcohols at 25 °C was used to synthesize amphiphilic block copolymers of poly(ethyl lactate acrylate)-b-poly(glycerol acrylate). Unfortunately, only micellae formation was studied in this work and tensile characteristics were not measured. Therefore, lactate-derived polyacrylates have potential in applications requiring elastomeric properties, but their mechanical properties have not been sufficiently studied and require careful investigation.

Itaconic acid is obtained using the fermentation technique and can be easily converted into various derivatives. Due to the presence of a double bond in its structure, itaconic acid can be polymerized by free-radical polymerization, including emulsion methods. Novel elastomer poly(diethyl itaconate-co-butyl acrylate-co-ethyl acrylate-co-glycidyl methacrylate) was obtained by redox emulsion polymerization [167]. Composite materials with carbon black were studied. For block-copolymer containing 10 wt.% of diethyl itaconate, the following tensile parameters were estimated: tensile strength of 14.5 MPa and elongation at break of 305%. These properties were compared to commercially available acrylate rubber AR72LS (see Table 8).

Itaconic acid derivatives—imides and esters—were used in RAFT polymerization to obtain block copolymers with soft poly(itaconate) block and hard poly(itaconimide) block [168]. Microphase separation was studied by means of atomic force microscopy, and lamellar and cylindrical morphologies were found. Based on these findings and dynamic mechanical properties, the typical elastomeric behavior of copolymers was stated. Itaconic acid was used in another study [169], along with renewable glycidyl methacrylate and succinic acid, as a source of biobased di- or tri-functional acrylates. Then, these sustainable monomers were polymerized by means of digital light processing without diluters. The obtained polymers were characterized by a dense cross-linked network and, consequently, rigid tensile behavior. Very low values of elongation at break (0.84–3.42%) and high values of elastic modulus (1563–4480 MPa) and tensile strength (31.1–45.2 MPa) were estimated. In this particular case, very rigid copolymers were synthesized, which cannot be considered as soft elastomers, but in broad terms, this approach is very promising for obtaining photoactive acrylates for 3D printing. Presumably, the decrease in the cross-linking degree may result in a softer elastomer with better tensile properties.

Glucose can be converted to acrylate derivative by direct modification. This monomer was used as a glassy component for developing elastomers and adhesive materials. Di- and triblock copolymers with n-butyl acrylate were synthesized by RAFT polymerization [170]. The authors pointed out that triblock copolymers demonstrated excellent thermomechanical and adhesion properties but moderate elastomeric properties: elongation at break was 171%, and tensile strength was 0.573 MPa. To improve the mechanical properties, the optimization of the block length ratio was suggested.

Biobased copolymers with a complex architecture were developed by [171]. The rigid backbone composed of chitin and grafted side chains consisted of lignin-derived poly(vanillin methacrylate) as hard segments and plant-oil-based poly(lauryl acrylate) as soft segments. Additionally, renewable 1,5-diaminopentane was used to create dynamic imine bonds. For different copolymer compositions, the following ranges of tensile strength, elongation at break, and elastic modulus were obtained: 0.5–6.1 MPa, 176–2781% and 2.7–39.3 MPa. Feed ratio and chitin content play important roles in the mechanical performance of copolymers. Another example of using lignin as a source for polyacrylate is described in [172]. Linear ABA-type triblock copolymers with lignin-derived poly(guaiacyl acrylate) glassy end blocks and polymethylacrylate (PMA) soft middle block was obtained by SET-LRP. Copolymers with different lengths of the middle block were studied. With the increase in the PMA block length, the tensile strength can be enhanced from 3.9 to 8.1 MPa, and elongation at break from 279 to 541%.

Polymethylmethacrylate derived from rosin was used as sustainable hard end blocks in triblock copolymers, with polybutylacrylate as the soft middle block [173]. Polymerization was carried out using the ATRP technique. Mechanical properties were measured for one sample with optimal composition and well-defined cylindrical morphology. A traditional fossil-derived acrylic copolymer was synthesized to compare properties (see Table 9).

Soybean oil (meth)acrylates were copolymerized with styrene using the ATRP method yielded in ABA-type triblock copolymers [174]. Bis-Triazolinedion (bis-TAD) was used for click-reaction to create bridges between soft middle blocks because they contained double bounds, which allowed for this type of click-coupling. This makes it possible to increase the tensile strength without the deterioration of elongation. Cyclic tensile tests showed excellent elastic recovery characteristics. With an increase in PS fraction, the tensile strength can be changed from 0.9 to 6.6 MPa; elongation at break was not affected by this parameter and was equal to approximately 100% for all samples. With an increase in poly(soybean acrylate) content, elongation at break can be enhanced from 60 to 189%. The addition of only 1 mol.% of bis-TAD resulted in two-times higher tensile strength, while elongation at break was maintained.

## 5. Alternative Types of Elastomers

In the previous sections, approaches to the green synthesis of three types of elastomers widely used for soft actuators development were discussed. Here, alternative types of biobased elastomers and their properties will be considered. Their possible use as a base material for soft actuators will also be mentioned.

**Figure 13:**
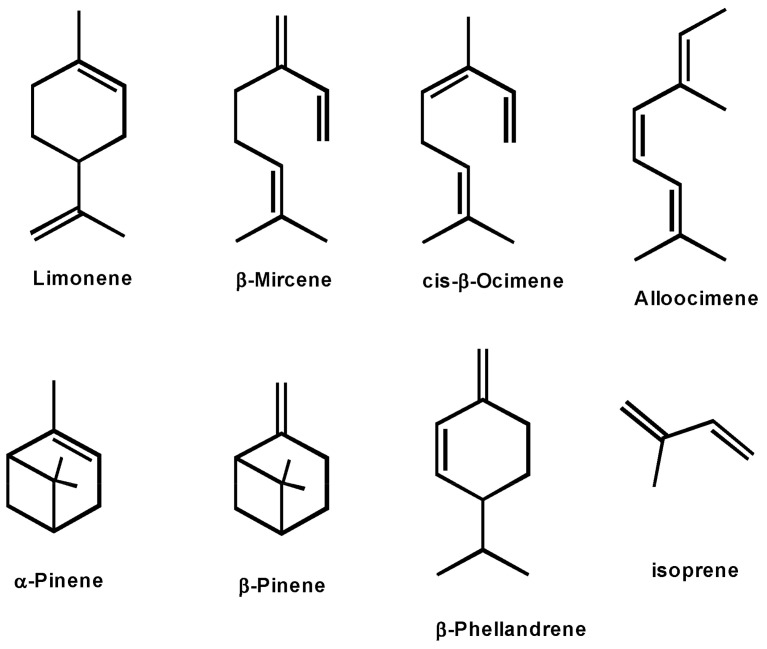
Common terpenes and terpenoids (adapted from [176]).

The first large group of biobased elastomers is terpene-based polymers [175,176]. Terpenes are an important class of biomass-derived compounds with an impressive range of chemical structures (see Figure 13). The most studied monomers are β-myrcene, β-pinene, limonene, and β-farnesene. Terpenes can also be chemically transformed into new renewable monomers, for example, by means of thiol−ene click chemistry [177,178]. Terpenes formally consist of isoprene units; therefore, they represent a promising monomer tool kit for anionic polymerization. One of the most used monoterpenes—β-myrcene—naturally occurs in different plants, but direct extraction is not cost efficient, so it is produced on large scales from turpentine oil. Another popular terpene—β-farnesene—is produced by Amyris Inc. from Brazilian sugar cane in an engineered yeast fermentation process [179]. Living anionic polymerization of terpenes is described for 1,3-diene monomers β-myrcene- and β-farnesene [180,181]. The chemical structure of polymyrcene and polyfarnesene is similar to natural rubber, which makes them very promising biobased alternatives for the design of sustainable elastomeric materials that will be able to compete with commercial fossil-based ones. One more step towards fully sustainable elastomers can be made by performing anionic polymerization of dienes in “green” solvents, such as 2-methyltetrahydrofuran or limonene [182,183].

Many studies are devoted to the synthesis of elastomers from terpenes, especially from β-myrcene and β-farnesene. Most synthetic approaches use anionic polymerization; however, some studies have focused on emulsion polymerization [184], which is more preferable due to its higher sustainability.

Preliminary studies on the mechanical properties of soft rubber obtained from myrcene by emulsion polymerization showed a tensile strength of 4.14 MPa and elongation at break of 300% [185]. Then, different synthetic approaches were developed [186,187], but no data were obtained on the mechanical properties of the elastomers produced. Further research was devoted to different terpene-based copolymers, such as poly (α-methyl-p-methylstyrene-b-myrcene-b-α-methyl-p-methylstyrene), which exhibited tensile strength up to 10 MPa and elongation at break up to 1300% [188]. In order to replace butadiene in traditional styrene-butadiene rubber, styrene-myrcene-butadiene rubber was obtained with a maximum tensile strength of 12.60 MPa and elongation at break of 620% [189]. The synthesis of β-myrcene/isobornyl methacrylate-based triblock copolymers, synthesized by means of nitroxide-mediated controlled radical polymerization, was reported [190]. These copolymers exhibited a tensile strength of 4 MPa and elongation at break of 500%. Among others, terpene-based copolymer rubbers with ocimene showed improved mechanical properties (stress at break = 17.81 MPa and elongation at break = 645%).

The next promising sustainable class of elastomers is represented by materials based on itaconic acid and its derivatives. The possibility of obtaining methacrylic acid from itaconic acid for biobased acrylic elastomers production was discussed in the previous section, but its potential is significantly wider and plenty of other biobased monomers can be derived from it.

**Figure 14:**
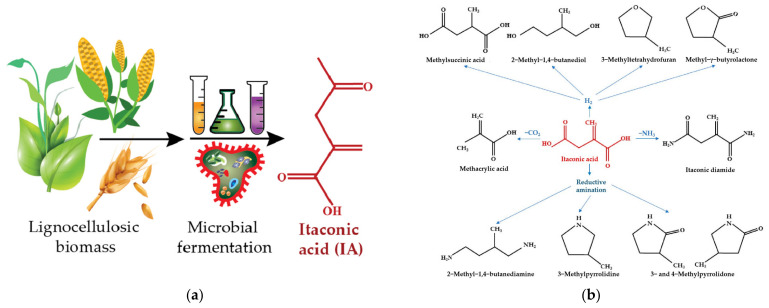
(**a**) Production of itaconic acid; (**b**) Some important derivatives of itaconic acid [191].

Industrially, the production of itaconic acid [191] is performed through the fermentation (see Figure 14a) of lignocellulosic biomass with fungi such as Aspergillus terreus and Ustilago maydis strains. As an alternative method for metabolical engineering, bacteria such as Escherichia coli and Corynebacterium glutamicum can be used. Biobased itaconic acid and its derivatives can replace many petrochemically-produced monomers due to their multifunctional structure (see Figure 14b). It was shown that biobased polyester elastomers can be synthesized from itaconic acid [192]. Another example is sustainable poly(dibutyl itaconate-co-butadiene) elastomers with curable double bonds, which were synthesized by emulsion polymerization of biobased dibutyl itaconate with butadiene [193]. The mechanical properties of this elastomer are comparable with those of traditional rubbers: the values of stress and elongation at break were over 2 MPa and 600%, respectively. This makes said elastomers good possible substitutes for conventional synthetic elastomers based on fossil resources. Solventless emulsion polymerization is also a big advantage of this methodology, meeting the requirements of green chemistry.

The wide group of materials includes elastomers based on lactides and lactones [194]. p-Cresol is a component of lignin bio-oils and can be used as the starting material in the sustainable production of γ-methylcaprolactone (γMCL) [195]. This method proved its efficiency and economic benefits. Nevertheless, this technology still does not meet the requirements of green chemistry principles, and new routes for the sustainable synthesis of lactones are being developed.

**Figure 15:**
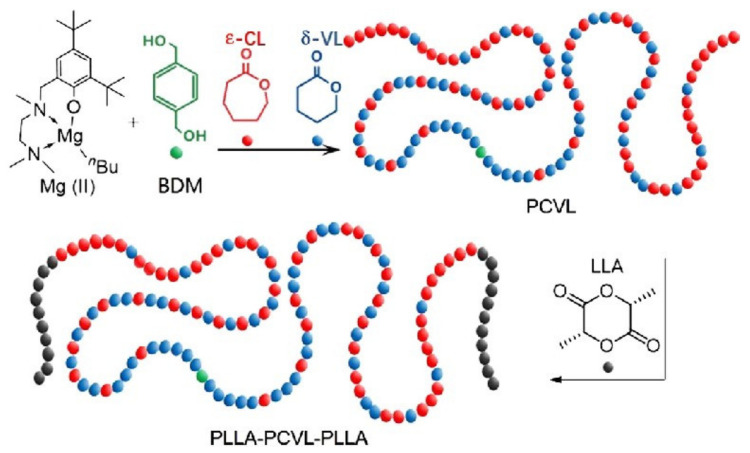
Synthesis of triblock co-polyester TPEs PLLA−PCVL−PLLA by living/controlled Mg(II)/BDM catalyst system [196].

Numerous studies are devoted to the synthesis of polyester elastomers with enhanced mechanical properties. Triblock polyester elastomers consisting of poly(L-lactide) as hard segments and poly(ɛ-caprolactone)-co-poly(δ-valerolactone) as soft segments were obtained by living ring-opening polymerization of cyclic lactones at room temperature (see Figure 15) [196]. The mechanical properties of block copolymers can be tuned by adjusting their composition. High stretchability up to 2100% and tensile strength up to 71.5 MPa were estimated.

The effect of cross-linking by bis(b-lactone) on the properties of well-defined star-shaped poly(γ-methyl-ε-caprolactone) was studied by Guilhem X. De Hoe et al. [197]. The different initial molecular weight of the polymer resulted in elongation at break in the range of 100–900%, and tensile strength in the range of 1–6 MPa. It was concluded that the tensile properties of the obtained elastomer with longer segments between cross-links were competitive with commercial polyisoprene-based rubber.

**Figure 16:**
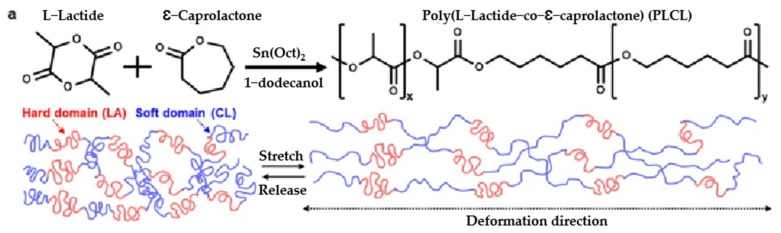

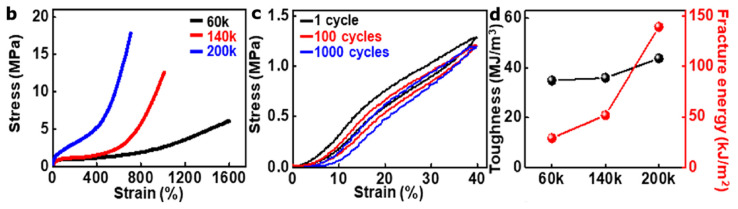
(**a**) Polymerization scheme; (**b**–**d**) mechanical characteristics of poly(L-lactide-co-ε-caprolactone) elastomers [199].

Different architectures of polyester-based copolymers can be obtained by different polymerization techniques. Triblock copolymers poly(styrene-b-(ε-caprolactone-co-4-methyl-ε-caprolactone)-b-styrene) were obtained by two-stage polymerization [198]. First, the soft middle blocks of poly(ε-caprolactone-co-4-methyl-ε-caprolactone) were synthesized by ring-opening polymerization. Then, the ATRP method was used to obtain hard end blocks of polystyrene. Elongation at break was found to be up to 1100%, and tensile strength up to 42 MPa. Graft copolymers based on a PLLA-macromonomer with grafted chains of poly(γMCL) with varied lengths of backbone and grafted chains, and with different grafting density, showed a very wide range of tensile modulus: from 0.7 to 148 MPa, and high elongation at break up to 2170%. A recent article in *Nature Communication* [199] describes the synthesis of ultra-stretchable and biodegradable poly(L-lactide-co-ε-caprolactone) elastomers and the fabrication of smart, soft actuators and suture-free cardiac-integrated electronics based on this sustainable material. The molecular weight of a polymer affects its mechanical properties, which can vary greatly: tensile strength values from 5 to 20 MPa and elongations at break from 700 to 1600% (see Figure 16).

Recent advances in aerobic fermentation have made it possible to obtain aminoacids of high-purity at a relatively low price. However, the synthesis of elastomers from aminoacids is understudied. A new method of polylysine synthesis from lysine by ring-opening polymerization was suggested [200]. The resulting polymers were characterized in elongation at break of 500–700% and a tensile strength of 10–15 MPa. Another example is grafting of histidine onto poly(oleic acid), followed by crosslinking to produce self-healing elastomers.

Plant oils, such as soybean or castor oils, can also serve as an efficient bio-source for the development of fully biobased elastomers. Furan functionalization of castor oil using one-pot solventless ring-opening polymerization from renewable resources was described in [201]. Subsequent cross-linking was performed by Diels-Alder cycloaddition, using an aromatic bismaleimide and a fatty acid-based bismaleimide. It was shown that a wide range of mechanical properties could be reached by changing the synthesis conditions. Young’s modulus was in the range of 0.5–20 MPa with elongation at break up to 487%. The recovery of 100% fracture strain was achieved within seconds at room temperature, and a capability of self-healing at room temperature was demonstrated. It is worth noticing that in this study, the obtained elastomer was successfully used as part of a soft pneumatic gripper.

## 6. Conclusions and Perspectives

Green chemistry is a rapidly developing area that is now gaining significant attention. The polymer industry is extremely huge and is still strongly dependent on petroleum products. The main polymeric materials are still produced by traditional methods based on fossil raw materials. However, issues of safety for the environment and human health are becoming more acute. In this regard, methods for obtaining various monomers from bioresources are also rapidly developing, and such monomers are becoming cheaper and more accessible.

In terms of the practical applications of polymers, an extremely popular area now is the development of soft robots, which implies the creation of soft actuators. These actuators have interesting applications in medicine, such as in the creation of soft robotic systems for surgery and soft 3D bioprinting with a high degree of freedom, which can directly deliver biomaterials into defect tissue. Within this field, one promising area is the development of DEAs that consist of elastomers as the main building material. Silicones, acrylic elastomers, and polyurethanes have been proven to be the most efficient materials due to their excellent electromechanical properties. Similar to other polymer production, the elastomer market is based entirely on petroleum products. Among the three classes of elastomers mentioned, polyurethanes have always been of the greatest concern because they contain toxic isocyanates, which are in turn produced with the use of extremely poisonous phosgene gas. Silicones and acrylic elastomers do not contain such hazardous components, but their synthesis still does not meet the principles of green chemistry because it involves non-sustainable reagents and often requires inefficient reaction conditions.

A wide variety of bioresources have been applied to produce monomers for the synthesis of elastomers, such as terpenes, vegetable oils, glucose, lignin, rosins, etc. At present, the production of many of them is quite cost-effective. A large number of scientific studies are devoted to the synthesis of elastomers from bioresources. Many types of raw materials have been tested, various methods have been used, and different structures and compositions have been investigated. Regarding the synthetic methods themselves, many of them imply the use of reagents that are not environmentally friendly, such as solvents, catalysts, and initiators. Although the elastomer itself is fully biobased, the synthetic process cannot be considered sustainable.

The mechanical properties of elastomers are very important characteristics that determine the potential use of the material in various applications. In many publications, mechanical parameters such as tensile strength, elongation at break, and Young’s modulus have been determined, and an evaluation of whether the mechanical properties of bioelastomers can compete with those of petroleum-based ones has been made, although it should be noted that in several studies that are very promising from a synthetic point of view, these parameters have not been determined.

There are only a few studies in which the electrical properties of bioelastomers have been measured. These material properties are also very important in the development of soft actuators. Moreover, new synthetic routes and a completely different composition of the elastomer could contribute, for example, to increasing the dielectric permittivity of the material, which is an important parameter that affects the actuation voltage. Furthermore, in general, these investigations would not only reveal the advantages but also the possible disadvantages of bioelastomers and would serve as an encouragement for further development in this field.

There is also a serious lack of studies where bioelastomers would be practically applied to the creation of soft robots, although the mechanical properties established for synthesized bioelastomers would allow such experiments.

In terms of green chemistry in relation to elastomer synthesis, the prospects for development and research are consistent with the main direction in the field of sustainable polymer production:

- Complete elimination of toxic reagents and maximal use of bio-materials, not only for monomers but also for all reaction components.
- The development of synthetic methods carried out in milder conditions.
- Biodegradability.
- Recyclability.

The advantages and challenges of existing “green” methods of elastomer synthesis are summarized in Table 10.

The development of methods that enable the polymerization of biomonomers and provide precise control of the structure of the resulting polymer is also very important.

Based on the reported data, one can conclude that today, a comprehensive study on the electromechanical properties of obtained elastomers and their practical application for creating soft actuators is essential to the already well-developed synthetic trend because these aspects are currently extremely scarce. Furthermore, since the green chemistry of elastomers and the development of soft robots are two rapidly developing areas, their integration is inevitable and will become more real over time.

## Figures and Tables

**Table 1:** Typical components in the manufacturing of PU (adapted from [78].

Component	Type	Compound
Diisocyanates	Aromatic	Toluene-2,4/2,6-diisocyanate (TDI)
		4,4′-methylene-bis-(phenylisocyanate) (MDI)
	Alicyclic	Isophoronediisocyanate (IPDI)
		4,4-methylene-bis(cyclohexylisocyanate)
	Aliphatic	Hexamethylene-1,6-diisocyanate (HDI)
Polyols	Aromatic polyethers	Dianol 24
	Aliphatic polyethers	Polyethylene oxide Polypropylene oxide Poly(tetramethylene oxide) glycol
	Aliphatic polyesters	Polyadipates of ethylene glycol Diethylene glycol Polycaprolactonediol
	Polycarbonates	1,6-hexanediol polycarbonate diol
Chain extenders	Diols	Ethylene glycol 1,3-propanediol 1,4-butanediol
	Diamines	1,2-ethylenediamine 1,6-hexamethylenediamine
Catalysts	Amine	1,4-diazabicyclo-[2,2,2]-octane
	Tin	Dibutyltindilaurate

**Table 2:** Properties of PUs obtained in [88].

Sample	E, MPa	Tensile Strength, MPa	Elongation at Break, %
HDI-FLO	80.1 ± 10.5	29.3 ± 0.7	813 ± 8
MDI-FLO	19.6 ± 1.9	26.0 ± 3.1	454 ± 18

**Table 3:** Mechanical properties and hardness of PUs [94].

Sample ^1^	Tensile Strength, MPa	Elongation, %	Shore A
HP-2.0	3.29 ± 0.02	1718.6 ± 8.6	59
HP-2.5	7.34 ± 0.04	450.7 ± 2.3	78
HP-3.0	23.45 ± 0.12	528.7 ± 2.6	84
MP-2.0	30.69 ± 0.15	977.8 ± 4.9	71
MP-2.5	10.07 ± 0.05	698.4 ± 3.5	73
MP-3.0	11.07 ± 0.06	489.9 ± 2.5	79

^1^ Sample abbreviation is composed according to the following pattern: type of isocyanate used (“HP” for H_12_MDI and “MP” for MDI)—[NCO]/[OH] ratio.

**Table 4:** Mechanical properties and hardness of biobased PUs [95].

Sample ^1^	Tensile Strength, MPa	Elongation, %	Shore A
H-BDO-2.0	4.61	1316.80	70
H-BDO-2.5	7.48	1024.50	79
H-BDO-3.0	31.61	635.70	85
M-BDO-2.0	28.84	1080.90	76
M-BDO-2.5	31.42	1076.90	77
M-BDO-3.0	35.04	693.90	86

^1^ Sample abbreviation is composed according to the following pattern: type of isocyanate used (“H” for H_12_MDI and “M” for MDI)—type of chain extender—[NCO]/[OH] ratio.

**Table 5:** Typical properties of traditional polyurethane elastomers.

Material	Elongation at Break, %	Tensile Strength, MPa	Young’s Modulus, MPa
Dureflex A4700	500	37.9	3.4 (100% modulus)
TPUs [114]	390–580	46–54	-
TDI-based	600	31	-
MDI-based	600	54	-
PU bioelastomers [115]	100–950	4–60	7–70

**Table 6:** Properties of obtained samples compared with those of two commercial silicone elastomers.

Sample	Elongation at Break, %	Young’s Modulus, MPa	Ɛ_r_ (1 kHz)	Actuation Strain, %
Cl-5.5 ^1^ [136]	125	0.14	4.6	53
Cl-8.5 ^2^ [136]	75	0.07	5.2	61
Sylgard 186 [137]	600	0.7	2.8	1.8
Elastosil 2030 [136]	310	1.25	2.6	11

^1^ silicone elastomer with 5.5 mol.% of chloropropyl groups. ^2^ silicone elastomer with 8.5 mol.% of chloropropyl groups.

**Table 7:** Some typical mechanical properties of silicone elastomers.

Sample	Elongation at Break, %	Young’s Modulus, MPa	Ɛ_r_ (1kHz)	Hardness
Ecoflex 00–30	900	0.1	3.4	00–30
Sylgard 184 [138]	150	2.4	2.7	50 A
Sylgard 186	600	0.7	2.8	24 A
Elastosil 2030	310	1.3	2.6	27 A
Elastosil M4601	700	0.3	-	28 A

**Table 8:** Mechanical properties of obtained elastomers and commercially available AR72LS acrylate rubber [167].

Sample	Tensile Strength, MPa	Elongation at Break, %	Hardness Shore A
PDEBEG-50	8.4 ± 0.2	249 ± 13	72 ± 1
PDEBEG-40	10.8 ± 0.2	280 ± 10	69 ± 1
PDEBEG-30	11.4 ± 0.3	234 ± 14	76 ± 2
PDEBEG-20	12.7 ± 0.3	345 ± 27	63 ± 1
PDEBEG-10	14.5 ± 0.4	305 ± 23	63 ± 1
PDEBEG-0	13.8 ± 0.3	346 ± 25	60 ± 1
AR72LS	13.8 ± 0.2	285 ± 6	66 ± 1

**Table 9:** Tensile properties of DnBD33 (biobased) and MnbM35 (fossil-based) triblock copolymers [173] (adapted with permission from [Ding, W.; Wang, S.; Yao, K.; Ganewatta, M.S.; Tang, C.; Robertson, M.L. Physical Behavior of Triblock Copolymer Thermoplastic Elastomers Containing Sustainable Rosin-Derived Polymethacrylate End Blocks. *ACS Sustain. Chem. Eng.* **2017**, *5*, 11470–11480]. Copyright {2017} American Chemical Society).

Triblock Copolymer	Elongation at Break, %	Tensile Strength, MPa	Young’s Modulus, MPa
DnBD33	242 ± 8	1.48 ± 0.07	0.75 ± 0.03
MnBM35	283 ± 29	4.06 ± 0.31	2.5 ± 0.5

**Table 10:** Advantages and challenges of existing “green” methods of elastomer synthesis.

Advantages	Challenges
Minimization of environmental hazards Prevention of occupational risks Safety for final product users Economic benefits Switch from petroleum-based products to renewable sources Wide range of biobased monomers for developing new elastomers Possibility of degradable material design	Possible deterioration of material properties Commercial unavailability of some biobased monomers Lack of investigations concerning mechanical and especially electrical properties of elastomers Not fully sustainable synthetic process

## Data Availability

Not applicable.
